# Health-related quality of life and post-traumatic stress disorder symptoms in accident and emergency attenders suffering from psychosocial crises: a longitudinal study

**DOI:** 10.1111/j.1365-2648.2011.05752.x

**Published:** 2012-02

**Authors:** Mette Senneseth, Kjersti Alsaker, Gerd Karin Natvig

**Affiliations:** Mette Senneseth MSc RN Psychiatric Nurse Department of Public Health and Primary Health Care, University of Bergen, Norway and Bergen Accident and Emergency DepartmentBergen, Norway; Kjersti Alsaker PhD RNSenior ResearcherUni Health, National Centre for Emergency Primary Health CareBergen, Norway; Gerd Karin Natvig PhD RNProfessorDepartment of Public Health and Primary Health Care, University of BergenNorway

**Keywords:** Accident and Emergency department, acute psychosocial crisis intervention, health-related quality of life, post-traumatic stress disorder symptoms, psychiatric nurses, psychosocial crisis

## Abstract

**Aims:**

This paper is a report of a study of health-related quality of life and post-traumatic stress disorder symptoms in patients attending an Accident and Emergency department because of psychosocial crises.

**Background:**

Psychosocial crises are commonplace globally, but there is little knowledge about patients attending Accident and Emergency departments because of psychosocial crises.

**Methods:**

Data were collected at an Accident and Emergency department in Norway from September 2008 to June 2009. A total of 99 adults participated in the baseline study and 41 of these participated at 2 months follow-up. The Short Form-36 Health Survey and the Post Traumatic Symptom Scale were used to obtain data.

**Findings:**

Participants reported significantly lower scores in all health-related quality of life domains at baseline compared with the general Norwegian population. The mental health score was two standard deviations below the norm. Health-related quality of life scores were improved and post-traumatic stress disorder symptoms were reduced after 2 months. High levels of post-traumatic stress disorder symptoms were reported by 78% of the participants at baseline and 59% at follow-up. Participants with high levels of post-traumatic stress disorder symptoms at follow-up also reported low health-related quality of life scores.

**Conclusion:**

This study suggests a need for an acute psychosocial intervention and an opportunity to receive follow-up support at Accident and Emergency departments.

## Introduction

People all over the world experience psychosocial crises in their lives (Bonanno 2004). Some seek professional help at Accident and Emergency departments (A&Es) (Keene & Rodriguez 2007, Try *et al.* 2008, Zakariassen *et al.* 2010). Psychosocial crises can be initiated either by psychosocial problems (Kelleher *et al.* 2000) [e.g. mental health (MH) problems, difficult social situations or severe family problems] or by traumatic life events (e.g. physical or sexual assault or bereavement) that induce psychological symptoms. Psychological symptoms frequently occur after traumatic life events (Bisson *et al.* 2007). Most people do not develop a problematic psychological response following trauma, or, if they do, they recover without any professional help (Bonanno 2004, Bisson *et al.* 2007). However, some people develop psychological difficulties that require therapeutic intervention (Wang *et al.* 2005, Bisson *et al.* 2007, Roberts *et al.* 2009). There is little knowledge internationally about the health of people attending A&Es because of psychosocial crises and their need for psychosocial support. Such knowledge may be essential for the intervention that is offered these people.

### Background

Over the past decades, people’s quality of life (QOL) has become increasingly important in health care and health research. The term ‘health-related quality of life’ (HRQoL) narrows the focus to the effects of health, illness and treatment on QOL (Ferrans *et al.* 2005). HRQoL is a multidimensional concept that consists of physiological, psychological and functional aspects of well-being as seen from the individual’s own perspective (Kvarme *et al.* 2009). A number of studies have shown that traumatic life events have a negative impact on HRQoL, such as physical and sexual assault (Sadler *et al.* 2000), exposure to domestic violence (Alsaker *et al.* 2006), traffic-related injuries (Wang *et al.* 2005), critical illness (Deja *et al.* 2006), sexual abuse (Dickinson *et al.* 1999) and military combat (Schnurr *et al.* 2006, Richardson *et al.* 2008). In four of these studies (Wang *et al.* 2005, Deja *et al.* 2006, Schnurr *et al.* 2006, Richardson *et al.* 2008), poor HRQoL is associated with post-traumatic stress disorder (PTSD), which is an anxiety disorder (Roberts *et al.* 2009). Schnurr *et al.* (2006) found that changes in PTSD symptoms are related to changes in HRQoL and a negative correlation between PTSD and QOL was found by Wang *et al.* (2005). Other psychosocial crises such as partnership splits and exposure to non-domestic violence have also been associated with poor MH and low QOL. However, in these studies different health- and quality-of-life questionnaires were used. Using the General Health Questionnaire (GHQ-12), Willitts *et al.* (2004) found that partnership splits had a negative impact on MH. In a Norwegian study, Johansen *et al.* (2007) found that a high level of PTSD symptoms in victims of non-domestic violence was associated with low QOL, measured by the World Health Organization’s Quality of Life questionnaire (WHOQOL-BREF). Only four of these studies measured the acute effect of the psychosocial crisis on MH (Willitts *et al.* 2004), QOL (Johansen *et al.* 2007) and HRQoL (Wang *et al.* 2005, Alsaker *et al.* 2006).

In a study of A&E contacts in Norway, diagnoses related to psychiatric illness were found in 2·7% of all events and the most frequently diagnosed subgroups were depression/suicidal behaviour, anxiety and substance abuse (Johansen *et al.* 2009). In another study, psychosocial crises (psychiatric illnesses and harmful events) were found in 3·7% (Zakariassen *et al.* 2010). A study of a psychosocial crisis support team at an Norwegian A&E reported that people contacted the team because of life crises such as serious life events, worries about others and interaction problem in the family in 65·5% (*n* = 901) of the events and that symptom of psychiatric illness (such as depression/suicidal behaviour, anxiety and substance abuse) were found in 29·6% (Try *et al.* 2008). However, we found no longitudinal studies that had examined HRQoL and PTSD symptoms in people suffering from acute psychosocial crises attending A&E.

## The study

### Aim

The aim of the study was to examine HRQoL and PTSD symptoms in people attending an A&E because of psychosocial crises.

### Design

This was an observational longitudinal study as stated by Polit and Beck (2008).

We hypothesized that:

- People who seek help at A&E, suffering from psychosocial crises, have lower HRQoL than the general population at presentation and they have improved HRQoL after 2 months.
- These people have high levels of PTSD symptoms at presentation and they have reduced PTSD symptoms after 2 months.
- High levels of PTSD symptoms are linked to low HRQoL scores at follow-up.

### Location

The location for this study was in a psychosocial crisis support team at an A&E in the centre of a city in Norway with approximately 240,000 inhabitants. The A&E has a total of near 100,000 contacts annually (Bergen A&E Annual report 2009). The psychosocial crisis support team at the A&E has approximately 2000 contacts annually and about as many patients contact the psychosocial support team by telephone as by personal meeting (Try *et al.* 2008). The psychosocial crisis support team is on duty 7 days a week and is staffed with trained psychiatric nurses. The psychosocial crisis support team is meant to give acute help in a crisis. The personnel are meant to have competence in basic diagnostic categories and half of staff is trained in cognitive behavioural therapy (CBT). The psychosocial crisis support team consultations at the A&E include information about normal reactions to trauma, advice for coping with the situation, motivation by focusing on the person’s own ability to cope and emphasizing the importance of social support from close family and friends, as recommended by Bisson *et al.* (2007). Referral from the team to General Practitioner (GP) is provided in more than 50% of contacts (Try *et al.* 2008).

### Participants

People who attended the psychosocial crisis support team were asked about participation immediately after their first consultation with a psychiatric nurse in the team. The inclusion criteria were people who, during the recruitment period, were

- attending A&E because of a psychosocial crisis and who consulted a psychiatric nurse.
- 18 years or older.
- able to read and understand Norwegian.

People were offered three to four consultations with the support team. Persons with suicidal thoughts or psychiatric illnesses in need of therapy, were in addition referred to the medical department to see a doctor. Persons with psychotic symptoms were not offered contact with a psychiatric nurse in the team, but with a psychiatrist for immediate treatment. A total of 113 people were asked to take part in the study. Ninety nine participated in the baseline study and 41 also participated in the follow-up study.

### Data collection

Data were collected using a self-report questionnaire that participants confidentially filled out at the A&E. A new questionnaire was sent 2 months later to the participants who had accepted participation in the follow-up study. Data were collected from September 2008 to June 2009. Questionnaires were treated confidentially and neither name nor date of birth was recorded. Individual codes (birth year combined with the first letter of the person’s parents’ first names) were used for paired tests from baseline to follow-up. Reminders were sent only once. Both the baseline and the follow-up studies comprised questions about demographic data, PTSD symptoms and HRQoL.

### Instruments

#### The SF-36 Health Survey

The SF-36 Health Survey is a 36 item self-report questionnaire that assesses eight domains of physical and MH ranging from 0 to 100, where the highest score indicates optimal HRQoL and the lowest score indicates the poorest HRQoL. The eight domains are physical functioning (PF), role limitations because of physical health problems (role-physical, RP), bodily pain (BP), general health (GH) perceptions, vitality (VT), social functioning (SF), role limitations because of emotional problems (role-emotional, RE) and general MH (Ware & Sherbourne 1992). The first four domains (PF, RP, BP, GH) together constitute the physical health domains. The other four domains (VT, SF, RE, MH) constitute the MH domains. The SF-36 is widely used in health research and is validated and tested for reliability by several studies (Loge *et al.* 1998, Ware *et al.* 2002).

#### The PTSS-10

The Post-traumatic Symptom Scale (PTSS-10) is a 10 item self-report questionnaire that assesses the presence and intensity of PTSD symptoms (Mehlum & Weisæth 2002). Each of the 10 symptoms is rated on a 7-point Likert scale ranging from 1 (not at all/never) to 7 (very often) (Mehlum & Weisæth 2002). The 10 symptoms are sleeping problems, nightmares, feeling of depression, startle/jumpiness, isolation, irritation, mood swings, feelings of guilt/lowered self-esteem, fear of places and situations that remind the subject of the traumatic event and bodily tension (Eid *et al.* 1999). The PTSS-10 has been found to have good internal consistency and test–retest reliability (Eid *et al.* 1999) and to be a responsive, valid and reliable instrument in screening for PTSD (Stoll *et al.* 1999). The total score ranges from 10 to 70. A score of 35 or more justifies a PTSD diagnosis (Stoll *et al.* 1999).

### Ethical considerations

People were given both oral and written information about voluntary participation and that non-participation would not influence their treatment at the clinic. The Regional Committee for medical Research Ethics for Western Norway and the Norwegian Data Inspectorate approved the study.

### Data analysis

Data were processed and statistical analysis performed using spss 16·0 (Oslo, Norway) for Windows. SF-36 data were recoded and missing data replaced using the SF-36 health survey manual and interpretation guide (Ware *et al.* 2002). SF-36 data for the general Norwegian population were obtained from the Norwegian Coordinated Living Conditions Survey, cross-section 2002 (*n* = 5131), received from the Norwegian Social Science Data Services. The SF-36 results were then norm-based, meaning that results were standardized for age and gender according to the score of the general Norwegian population (2002), such that a value of 50 is the mean of the general Norwegian population and 10 is the standard deviation (Gandek *et al.* 2004, Magerøy *et al.* 2007, Alsaker *et al.* 2008). Norm-based scoring of SF-36 scales is recommended by developers because of the advantage of easily facilitating interpretation of results across measures, as all measures have comparable means and standard deviations (Gandek *et al.* 2004). A score of <50 shows a lower mean score than people of the same age and gender in the general Norwegian population. One-sample *t*-tests with a test value of 50 were used to compare means with the general Norwegian population, giving a *P* value for significantly different means if equal or <0·05, corresponding to the 5% significance level. A 95% confidence interval (CI) was used as an estimated statistical interval for the norm-based scores.

Paired samples *t*-tests were used to examine the changes in the SF-36 norm-based scores from baseline to follow-up (*n* = 41). The *P* value for change from baseline to follow-up indicates a statistically significant change in the norm-based SF-36 scores from baseline to follow-up if equal or <0·05.

The size of the changes in the norm-based SF-36 scores from baseline to follow-up was measured by the *effect sizes* (follow-up score minus baseline score divided by the standard deviation at baseline). Effect sizes were judged against standard criteria: trivial (<0·2), small (0·2 to <0·5), moderate (0·5 to <0·8) and large (≥0·8) (Cohen 1978).

Post-traumatic Symptom Scale-10 scores, measuring PTSD symptoms, were summarized for each participant both at baseline and at follow-up. Paired samples *t*-tests were used to examine the changes in the summarized scores from baseline to follow-up (*n* = 41). The *P* value for change from baseline to follow-up indicates a statistically significant change from baseline to follow-up if equal or <0·05. In addition, a cut-off score at 35 was used in accordance with other studies (e.g. Stoll *et al.* 1999, Deja *et al.* 2006) to categorize participants into two subgroups at 2 months follow-up; ‘PTSS-10 low-scoring’ (range 10–34) and ‘PTSS-10 high-scoring’ (range 35–70). PTSS-10 subgroups at 2 months follow-up were compared regarding the norm-based SF-36 mean scores reported by these participants, using independent samples *t*-tests for each of the eight SF-36 domains. Paired samples *t*-tests were used to find the change in norm-based SF-36 scores from baseline in PTSS-10 subgroups at 2 months follow-up.

## Findings

### Participants

**Figure 1:**
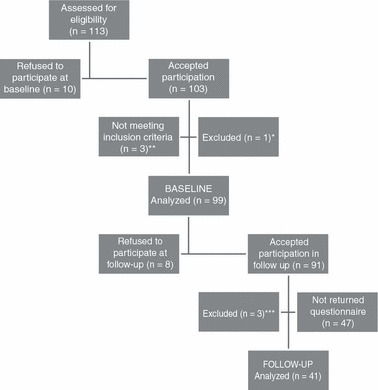
Flowchart. *One respondent was excluded because of almost blank questionnaire. **Three respondents completed questionnaire but were under 18 years of age and were excluded during processing of the data. ***Three respondents stated unknown personal codes at follow-up questionnaires and follow-up data could not be paired with baseline data.

In the baseline study, 113 people were asked to participate (Figure 1 flowchart). Ninety nine people were included, comprising 77 women and 22 men. Two months later 41 of these were included in the follow-up study, comprising 35 women and 6 men.

### Demographic data and reasons for attending A&E

The mean age (sd) was 33 years (13). More than half of participants were single or divorced (Table 1). Participants stated different reasons for attending the psychosocial support team at the A&E (Table 1). Severe difficulties related to their partner or a family member was the most frequent reason for attending the department (28·1%), followed by MH problems (26·3%) and sexual or physical assault (18·1%).

**Table 1:** Demographic characteristics and reasons for attending A&E at baseline (*n* = 99)

	*N*	%
Female	77	77·8
Male	22	22·2
Marital status		
Single	51	51·5
Married or living with partner	34	34·3
Girlfriend/boyfriend	7	7·1
Divorced	6	6·1
Not stated	1	1·0
Reasons to attend the A&E		
Severe difficulties related to partner or family member	28	28·1
Sexual or physical assault	18	18·1
Loss of loved one in sudden death or suicide	6	6·0
Other traumatic life event or psychosocial crisis	16	16·2
Mental health problems	26	26·3
Suicidal thoughts or plans	4	4·0
Not stated	1	1·0

A&E, Accident and Emergency department.

### Lost to follow-up

Participants who were lost to follow-up (*n* = 58) did not differ in any characteristics from the participants who were followed-up (*n* = 41), neither in age (*P* = 0·8), gender (*P* = 0·1), baseline PTSS-10 summarized scores (*P* = 0·2) nor in baseline HRQoL scores (*P* = 0·2–0·9).

### Number of consultations

At the follow-up after 2 months (*n* = 41), participants were asked about the total number of consultations they had had with a psychiatric nurse at the A&E. Twelve participants (29%) reported to have had four or more consultations, including the first consultation. Seventeen participants (42%) reported to have had two or three consultations, and 12 participants (29%) reported to have had only the first consultation.

### Health-related quality of life

#### Baseline (*n* = 99)

In raw scores from 0 to 100 the mean results (sd) for each domain were: PF 85·2 (21·9), RP 52·6 (42·2), BP 55·8 (27·6), GH 63·1 (23·2), VT 31·8 (19·8), SF 43·4 (27·2), RE 26·2 (38·4) and MH 39·4 (20·1).

Through standardized SF-36 results (norm-based scores), mean scores were compared with the mean of the general Norwegian population (in 2002), such that the mean of the general Norwegian population was 50 and 1 sd was 10. Participants in this study had lower norm-based scores in all eight HRQoL domains compared with the general Norwegian population at baseline (Table 2). The lowest norm-based scores were in MH (mean 26·9), SF (mean 30·9) and RE (mean 32·9).

**Table 2:** Health-related quality of life mean scores at baseline and at 2 months follow-up compared with the general Norwegian population

	Baseline (*n* = 99)		Follow-up (*n* = 41)			
			
SF-36 norm-based scores*	Mean (sd)	*P*†	Mean (sd)	*P*†	*P* value for change from baseline‡	Effect size§
Physical health domains						
Physical functioning (PF)	46·8 (12·1)	0·01	50·4 (7·0)	<0·01	0·18	0·2
Role-physical (RP)	41·5 (12·1)	<0·001	44·0 (12·8)	<0·01	0·40	0·2
Bodily pain (BP)	41·7 (10·7)	<0·001	46·4 (11·0)	0·04	0·01	0·4
General health (GH)	42·2 (10·9)	<0·001	43·7 (12·3)	<0·01	0·71	0·1
Mental health domains						
Vitality (VT)	37·3 (9·7)	<0·001	44·0 (11·1)	<0·01	0·001	0·7
Social functioning (SF)	30·9 (12·4)	<0·001	39·4 (14·1)	<0·01	0·001	0·6
Role-emotional (RE)	32·9 (11·9)	<0·001	38·5 (13·2)	<0·01	0·001	0·7
Mental health (MH)	26·9 (12·2)	<0·001	36·2 (14·9)	<0·01	0·001	0·7

* SF-36 results are standardized for age and gender according to the score of the general Norwegian population (2002), such that a value of 50 is the mean of the general Norwegian population and 10 is the standard deviation, as described in Gandek *et al.* (2004).

† *P* value for mean difference compared with the general Norwegian population found from one-sample *t*-test with a test value of 50. Significant difference if *P* value is <0·05.

‡ *P* value for change from baseline to follow-up (*n* = 41) found from paired samples *t*-tests. Significant change if *P* value is <0·05.

§ Effect sizes (the size of changes) are judged against standard criteria: trivial (<0·2), small (0·2 to <0·5), moderate (0·5 to <0·8) and large (≥0·8) (Cohen 1978).

#### Follow-up (*n* = 41)

After 2 months, the participants still had lower norm-based scores than the general Norwegian population (Table 2). However, the participants had improved their HRQoL in five domains from baseline to follow-up, including the four MH domains and BP (Table 2). On a standard scale consisting of ‘trivial, small, moderate and large’, the effect sizes show that the change in BP is judged as small, while the changes in the four MH domains are judged as moderate (Table 2).

### PTSS-10 scores

Mean PTSS-10 summarized score at baseline (*n* = 99), measuring the 10 PTSD symptoms: sleeping problems, nightmares, feeling of depression, startle/jumpiness, isolation, irritation, mood swings, feelings of guilt/lowered self-esteem, fear of places and situations that remind the subject of the traumatic event and bodily tension, was 44·7 (possible range 10–70; Table 3). Among the participants at baseline, 79% had a PTSS-10 total score greater than or equal to the cut-off score of 35 points, showing high levels of PTSD symptoms.

**Table 3:** PTSS-10 summarized score (possible range 10-70) measuring post-traumatic stress disorder symptoms at baseline and at 2 months follow-up

PTSS-10 summarized score		

Baseline mean score (sd) (*N* = 99)	Follow-up mean score (sd) (*N* = 41)	Change from baseline to follow-up* (*N* = 41)
44·7 (11·3)	37·2 (15·4)	*P* = 0·003

* *P* value found from paired sample *t-*test.

PTSS-10, Post-traumatic Symptom Scale-10.

At 2 months follow-up (*n* = 41), the mean summarized score for PTSS-10 was 37·2 (Table 3). PTSS-10 summarized scores were reduced from baseline to follow-up (*P* = 0·003). At follow-up, 59% of the participants had a PTSS-10 total score greater than or equal to the cut-off score of 35 points, showing high levels of PTSD symptoms, which can indicate a risk of developing PTSD.

### HRQoL and the PTSS-10 scores

**Figure 2:**
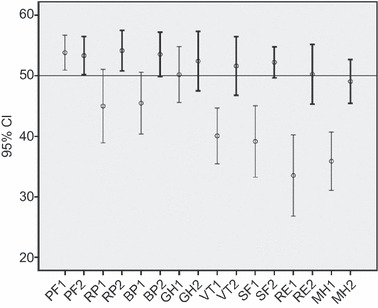
Health-related quality of life reported by participants with a Post-traumatic Symptom Scale (PTSS)-10 score below cut-off score at 35 at 2 months follow-up (*n* = 17). This figure presents mean score and 95% confidence interval (CI), based on approximate normal distributions, for the norm-based scores at baseline and 2 months later. The norm-based scores are the SF-36 scores standardized for age and gender according to the score of the general Norwegian population, such that the mean score of the general Norwegian population is 50 and standard deviation is 10 for all scales. PF, physical functioning; RP, role-physical; BP, bodily pain; GH, general health; VT, vitality; SF, social functioning; RE, role-emotional; MH, mental health; 1 = baseline; 2 = after two months.

**Figure 3:**
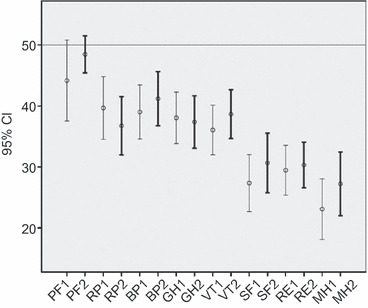
Health-related quality of life reported by participants with a Post-traumatic Symptom Scale (PTSS)-10 score greater than or equal to cut-off score at 35 at 2 months follow-up (*n* = 24). This figure presents mean score and 95% confidence interval (CI), based on approximate normal distributions, for the norm-based scores at baseline and 2 months later. The norm-based scores are the SF-36 scores standardized for age and gender according to the score of the general Norwegian population, such that the mean score of the general Norwegian population is 50 and standard deviation is 10 for all scales. PF, physical functioning; RP, role-physical; BP, bodily pain; GH, general health; VT, vitality; SF, social functioning; RE, role-emotional; MH, mental health; 1 = baseline; 2 = after two months.

Concerning the question if there is a link between PTSD symptoms and HRQoL, we looked at the differences in SF-36 scores between PTSS-10 subgroups in the follow-up study. The PTSS-10 high-scoring and low-scoring subgroups at 2 months follow-up differed in all HRQoL domains in the follow-up study (*P* < 0·001) (in PF *P* = 0·03). The PTSS-10 low-scoring people had improved HRQoL in six of eight domains (*P* < 0·01) (Figure 2). There was no statistically significant difference between the general Norwegian population and the PTSS-10 low-scoring people in the four MH domains and in GH after 2 months. PTSS-10 high-scoring people at 2 months follow-up reported lower HRQoL than the general Norwegian population in seven of eight domains (*P* < 0·001). For PF, we found no statistically significant difference (Figure 3). PTSS-10 high-scoring people at 2 months follow-up reported no statistically significant improvement in any HRQoL domain from baseline (Figure 3).

## Discussion

### Study limitations

A limitation of this study is that these findings cannot be generalized to people who experience psychosocial crises but do not attend an A&E. There may be a difference between people who attend A&Es because of psychosocial crises and those who experience psychosocial crises without seeking help. Another limitation is that we do not have data on whether participants were on medication or had taken alcohol, neither at presentation nor at follow-up. A previous study of people attending the psychosocial crisis support team at the A&E because of psychosocial crises showed that 4·9% reported substance abuse and 9·2% reported depression (Try *et al.* 2008). Depression and alcohol/substance abuse may have negative impact on HRQoL (Piccinni *et al.* 2006, Lahmek *et al.* 2009), while medications such as antidepressants can have a positive effect on HRQoL (Caruso *et al.* 2010). These variables should be included in further studies. A third limitation to the study is that we cannot through the statistical analyses used in this study measure the predictive value of the PTSS-10 instrument on HRQoL. And finally, more research is needed to be able to find reasons for the changes that take place in HRQoL and in PTSD symptoms. A randomized controlled trial with a control group is needed to investigate the effect of the psychosocial interventions that are given to this group.

### Health-related quality of life in A&E attenders suffering from psychosocial crises

Concerning our first hypothesis, participants reported lower HRQoL compared with the general Norwegian population in all eight HRQoL domains at presentation. The greatest differences were found in MH, SF and role limitations because of emotional problems. Participants reported almost as low scores in MH (mean 39·4) as abused women at Norwegian women’s shelters, who had a mean score of 35·6 (0–100 raw scores) (Alsaker *et al.* 2006). A study of women in the military who had experienced both sexual and physical assault, reported a mean score of 58·6 in MH (Sadler *et al.* 2000). However, assaults could have happened more than 10 years before, thus showing long-term effects on HRQoL (Sadler *et al.* 2000).

Wyrwich *et al.* (2005) emphasizes the importance of estimating clinically significant differences in QOL outcomes. In this study, the difference between participants and the general Norwegian population was clinically significant in the MH domain, with a difference of more than two standard deviations. The low scores in the MH domain reflect the persons feelings of nervousness and depression all of the time (Ware & Sherbourne 1992). A high score would reflect feeling peaceful, happy and calm all the time. These clinically significant findings imply that participants’ feelings of nervousness and depression, and the fact that they do not feel peaceful, happy and calm, could represent a risk of suicidal ideation (Wyrwich *et al.* 2005). This is supported by other studies showing that individuals experiencing negative and potentially traumatic life events are at increased risk for suicidal thoughts and behaviours (Kaplan *et al.* 1997, Dube *et al.* 2001). Concerning SF, it is well documented that social support is essential in people’s lives (Kendler *et al.* 2005). In personal crises, social support may be an important factor to recover (Deja *et al.* 2006, Bisson *et al.* 2007, McGrath *et al.* 2010). The low score in SF in our study reflects that it can be difficult for these people to apply for social support themselves. People suffering from psychosocial crisis therefore may need outreach help, which is suggested by other studies (Dyregrov 2001, McGrath *et al.* 2010). However, it is essential that social support is positive as negative social support may be harmful (Dyregrov 1990) and may increase the risk of developing PTSD (Brewin & Holmes 2003).

At 2 months follow-up, participants still reported lower HRQoL scores when compared with the Norwegian population in general. However, participants had higher scores at the follow-up in five HRQoL domains, showing that their MH and BP had improved from baseline. The improvements from baseline in VT, SF, RE and MH are by effect sizes judged to be clinically significant differences (Wyrwich *et al.* 2005). These improvements reflect that participants feel happier, more peaceful and calmer (MH) and that they have more energy (VT) after 2 months. In addition, they do not have the same limitations because of SF and emotional problems as they reported at baseline.

### Post-traumatic stress disorder symptoms

Concerning our second hypothesis, participants in the present study reported high levels of PTSD symptoms at presentation. Mean PTSS-10 summarized scores, measuring PTSD symptoms, were more than twice as high in participants in this study (44·7) as in Norwegian U.N. Peacekeepers (17·3) (Mehlum & Weisæth 2002). PTSS-10 scores improved from baseline to follow-up. These results show that PTSD symptoms decrease for people suffering from a psychosocial crisis in the 2 months after attending the A&E. These findings are supported by other studies that find that people can have psychological and behavioural reactions to traumatic events that diminish over time (Bonanno 2004, Bisson *et al.* 2007). Brewin (2003) claims that it may take 6–12 months before people who experience trauma no longer will have PTSD symptoms. A two-month follow-up, as used in our study, may according to this be a relatively short period of time to measure the decrease in such symptoms. This might explain why PTSS-10 mean scores still are relatively high at 2 months follow-up. However, people with high levels of PTSD symptoms after 3–4 months are likely to have chronic symptoms that will not improve further without intervention (Brewin 2003). A PTSD diagnosis cannot be given to participants in this study based on the PTSS-10 scores. An additional psychiatric evaluation is required and a specific prior traumatic event is a criterion. Nevertheless, the high PTSS-10 scores indicate severe psychological distress and reflect PTSD symptoms which are associated with poor HRQoL (Deja *et al.* 2006, Schnurr *et al.* 2006, Richardson *et al.* 2008).

### Health-related quality of life and the level of post-traumatic stress disorder symptoms

Concerning the question if there is a link between the level of PTSD symptoms and HRQoL in the follow-up study, we found that a high level of PTSD symptoms after 2 months was linked to lower HRQoL measures. These findings are supported by other studies (Deja *et al.* 2006, Schnurr *et al.* 2006, Richardson *et al.* 2008). In our current study, 59% of the participants at 2 months follow-up reported high PTSS-10 scores and they reported no statistically significant improvement in HRQoL from baseline. However, we do not know anything about neither the post-traumatic symptom scores nor HRQoL scores among participants in the time before the study. This could mean that some participants have had high post-traumatic symptom levels and low HRQoL both before and throughout the study. Depression, for example, can have a major negative impact on HRQoL (Goldney & Fisher 2004).

### Implications for practice and research

Findings in this study show that people attending an A&E experiencing psychosocial crises because of severe family problems, sexual or physical assault, bereavement or MH problems, have statistically significant impairments with respect to HRQoL and that they suffer from severe psychological distress. These findings suggest a need for an acute psychosocial crisis intervention. In addition, this study suggests that people who experience a psychosocial crisis may need outreach social support. A point for nursing staff to consider is to help these attenders to activate their social network and to make sure that they are not left to themselves when they leave the A&E.

In this study, participants had consultations with a psychiatric nurse in a psychosocial crisis support team at the A&E. We have not measured the effect of these consultations in this study. However, other studies emphasize the benefits of early psychosocial intervention (Dybdal 2001, Bisson *et al.* 2007, Dennis & Hodnett 2007) and social support (Deja *et al.* 2006, McGrath *et al.* 2010) in psychosocial crises. Findings in this study imply that many A&E attenders who are suffering from psychosocial crises will be in need of further therapeutic intervention. An appropriate intervention could be that people are offered a follow-up consultation with a psychiatric nurse 2 months after first attending an A&E, which includes screening for PTSD and secures the people adequate treatment. The PTSS-10 can be a useful supplemental instrument in identifying people who need further attention at presentation and in identifying people who are at risk of developing PTSD. People with PTSD should be referred to Trauma Focused CBT or eye movement desensitization and reprocessing treatment, both of which have been shown to be effective in treating this disorder (Bisson & Andrew 2007). However, a PTSD diagnosis should not be considered at presentation unless symptoms have lasted for at least 1 month, but should be considered at a follow-up consultation.

**What is already known about this topic**

- People suffering from psychosocial crises are commonplace globally.
- Nurses in Accident and Emergency departments often meet people suffering from psychosocial crises.
- Psychosocial crises may cause enduring mental health problems and impairments in health-related quality of life.

**What this paper adds**

- Health-related quality of life scores among people attending an Accident and Emergency department because of psychosocial crises are significantly low compared with the general population and the low mental health score is clinically significant.
- People attending an Accident and Emergency department because of psychosocial crises have high levels of post-traumatic stress disorder symptoms at presentation.
- High levels of post-traumatic stress disorder symptoms after 2 months are linked to low health-related quality of life scores.

**Implications for practice and/or policy**

- This study suggests that acute and follow-up psychosocial support at Accident and Emergency departments, that secures psychological treatment for people when indicated, should be available.
- The suicidal risk in people who suffer from psychosocial crisis should be assessed.
- Post-traumatic Symptom Scale-10 may be a useful instrument for psychiatric nurses when it comes to assessing post-traumatic stress disorder symptoms and to identify people in need for treatment.

Another important issue is the low MH scores in these people, which represent a possible risk of suicidal ideation. Assessing suicide risk may therefore be of crucial importance. Screening for suicide risk is provided by GP at A&E (Try *et al.* 2008), however, nurses can provide an opportunity for people to respond to this important question by asking, and secure that this question also is raised by the GP. A recent study (Hirsch *et al.* 2009) claims that suicidal outcomes in people experiencing negative or traumatic events are not inevitable, and finds that optimistic reframing of negative life events is associated with reduced suicide ideation. This suggests that early psychosocial interventions should be easy to get in touch with, and that a low threshold at A&Es for people experiencing a psychosocial crisis would be recommendable, such one could more easily identify people who need further psychological treatment. Nevertheless, further methodological research is needed to investigate the effect of early psychosocial crisis intervention on HRQoL and PTSD symptoms in people experiencing psychosocial crises attending an A&E. Concerning the limitation of this study on information about MH problems before presentation at A&E, The Mini-International Neuropsychiatric Interview (M.I.N.I.) which is a short but accurate structured psychiatric interview (Sheehan *et al.* 1998) would be appropriate and recommended in such research. Negative life events and poor social support is also associated with seasonal affective disorder (Michalak *et al.* 2003). These are interesting variables and may be included in further studies using Seasonal Patterns Assessment Questionnaire, the List of Threatening Experiences and the Oslo 3-Item Social Support Scale (Michalak *et al.* 2003).

## Conclusion

With regard to clinical implications, our results may be useful informing accident and emergency staff about the severe psychological distress psychosocial crises can lead to, and that the assessment of suicidal risk in these attenders is essential. One should ask these attenders if they have suicidal thoughts and immediately refer them to a medical doctor or a psychiatrist if they answer affirmatively to this. It also seems to be important to have an opportunity for follow-up to be able to identify people who need further treatment. In addition, nurses should help these attenders to activate their social network when leaving the A&E, as social support may be essential in recovering from a psychosocial crisis. The PTSS-10 may be a useful supplemental instrument to psychiatric nurses to identify persons who are in need of further intervention at presentation and those who are at risk of developing PTSD at follow-up. In terms of implications for policy, one should consider the need for an acute psychosocial intervention and an opportunity to receive follow-up support at A&Es. In terms of recommendations for future research, it would be appropriate to get more knowledge about the effect of acute psychosocial crisis intervention on PTSD symptoms and HRQoL given by psychiatric nurses to these attenders. Variables such as mental disorder and social support should be included in such research.

## References

[b1] Alsaker K, Moen B, Nortvedt MW, Baste V (2006). Low health-related quality of life among abused women. Quality of life research.

[b2] Alsaker K, Moen BE, Kristoffersen K (2008). Health-related quality of life among abused women one year after leaving a violent partner. Social Indicators Research.

[b3] Bergen A, E Annual report (2009). http://www.bergen.kommune.no/omkommunen/avdelinger/bergen-legevakt/919.

[b4] Bisson J, Andrew M (2007). Psychological treatment of post-traumatic stress disorder (PTSD). Cochrane Database of Systematic Reviews.

[b5] Bisson JI, Brayne M, Ochberg FM, Everly GS (2007). Early psychosocial intervention following traumatic events. The American Journal of Psychiatry.

[b6] Bonanno GA (2004). Loss, trauma, and human resilience. Have we underestimated the human capacity to thrive after extremely aversive events?. American Psychologist.

[b8] Brewin CR (2003). Posttraumatic Stress Disorder: Malady of Myth?.

[b9] Brewin CR, Holmes EA (2003). Psychological theories of posttraumatic stress disorder. Clinical Psychology Review.

[b10] Caruso R, Rossi A, Barraco A, Quail D, Grassi L, Italian FINDER study group (2010). The factors influencing depression endpoints. Research (FINDER) study: final results of Italian patients with depression. Annals of General Psychiatry.

[b11] Cohen J (1978). Statistical Power Analysis for the Behavioural Sciences.

[b12] Deja M, Denke C, Weber-Carstens S, Schröder J, Pille CE, Hokema F, Falke KJ, Kaisers U (2006). Social support during intensive care unit stay might improve mental impairment and consequently health-related quality of life in survivors of severe acute respiratory distress syndrome. Critical Care.

[b13] Dennis CL, Hodnett ED (2007). Psychosocial and psychological interventions for treating postpartum depression. Cochrane Database of Systematic Reviews.

[b14] Dickinson LM, deGruy FV, Dickinson WP, Candib LM (1999). Health-related quality of life and symptom profiles of female survivors of sexual abuse. Archives of Family Medicine.

[b15] Dube SR, Anda RF, Felitti VJ, Chapman DP, Williamson DF, Giles WH (2001). Childhood abuse, household dysfunction, and the risk of attempted suicide throughout the life span. Findings from the Adverse Childhood Experiences study. JAMA.

[b16] Dybdal R (2001). Children and mothers in war: an outcome study of a psychosocial intervention program. Child Development.

[b17] Dyregrov A (1990). Crisis intervention following the loss of an infant child. Bereavement Care.

[b18] Dyregrov A (2001). Early intervention – a family perspective. Advances in Mind–Body Medicine.

[b19] Eid J, Thayer JF, Johnsen BH (1999). Measuring post-traumatic stress: a psychometric evaluation of symptom and coping questionnaires based on a Norwegian sample. Scandinavian Journal of Psychology.

[b20] Ferrans CE, Zerwic JJ, Wilbur JE, Larson JL (2005). Conceptual model of health-related quality of life. Journal of Nursing Scholarship.

[b21] Gandek B, Sinclair SJ, Kosinski M, Ware JE (2004). Psychometric evaluation of the SF-36 Health Survey in Medicare managed care. Health Care Financing Review.

[b22] Goldney RD, Fisher LJ (2004). Double depression in an Australian population. Social Psychiatry & Psychiatric Epidemiology.

[b23] Hirsch JK, Wolford K, LaLonde SM, Brunk L, Parker-Morris A (2009). Optimistic explanatory style as a moderator of the association between negative life events and suicide ideation. Crisis.

[b24] Johansen VA, Wahl AK, Eilertsen DE, Weisaeth L, Hanestad BR (2007). The predictive value of post-traumatic stress disorder symptoms for quality of life: a longitudinal study of physically injured victims of non-domestic violence. Health and Quality of Life Outcomes.

[b25] Johansen IH, Morken T, Hunskår S (2009). Contacts related to psychiatry and substance abuse in Norwegian casualty clinics. A cross-sectional study. Scandinavian Journal of Primary Health Care.

[b26] Kaplan SJ, Pelcovitz D, Salzinger S, Mandel F, Weiner M (1997). Adolescent physical abuse and suicide attempts. Journal of the American Academy of Child & Adolescent Psychiatry.

[b27] Keene J, Rodriguez J (2007). Are mental health problems associated with use of Accident and Emergency and health-related harm?. European Journal of Public Health.

[b28] Kelleher KJ, McInerny TK, Gardner WP, Childs GE, Wasserman RC (2000). Increasing identification of psychosocial problems: 1979-1996. Pediatrics.

[b29] Kendler KS, Myers J, Prescott CA (2005). Sex differences in the relationship between social support and risk for major depression: A longitudinal study of opposite-sex twin pairs. The American Journal of Psychiatry.

[b30] Kvarme LG, Haraldstad K, Helseth S, Sørum R, Natvig GK (2009). Associations between general self-efficacy and health-related quality of life among 12-13-year-old school children: a cross-sectional survey. Health and Quality of Life Outcomes.

[b31] Lahmek P, Berlin I, Michel L, Berghout C, Meunier N, Aubin HJ (2009). Determinants of improvement in quality of life of alcohol-dependent patients during an inpatient withdrawal programme. International Journal of Medical Sciences.

[b32] Loge JH, Kaasa S, Hjermstad MJ, Kvien TK (1998). Translation and performance of the Norwegian SF-36 Health Survey in patients with rheumatoid arthritis. I. Data quality, scaling assumptions, reliability and construct validity. Journal of Clinical Epidemiology.

[b33] Magerøy N, Riise T, Johnsen BH, Moen BE (2007). Health-related quality of life in the Royal Norwegian navy: does officer rank matter?. Military Medicine.

[b34] McGrath P, Holewa H, McNaught M (2010). Surviving spousal bereavement – insights for GPs. Australian Family Physician.

[b35] Mehlum L, Weisæth L (2002). Predictors of posttraumatic stress reactions in Norwegian U.N. peacekeepers 7 years after service. Journal of Traumatic Stress.

[b36] Michalak EE, Wilkinson C, Hood K, Dowrick C, Wilkinson G (2003). Seasonality, negative life events and social support in a community sample. The British Journal of Psychiatry.

[b37] Piccinni A, Maser JD, Bazzichi L, Rucci P, Vivarelli L, Del Debbio A, Catena M, Bombardieri S, Dell’Osso L (2006). Clinical significance of lifetime mood and panic-agoraphobic spectrum symptoms on quality of life of patients with rheumatoid arthritis. Comprehensive Psychiatry.

[b38] Polit DF, Beck CT (2008). Nursing Research: Generating and Assessing Evidence for Nursing Practice.

[b39] Richardson JD, Long ME, Pedlar D, Elhai JD (2008). Posttraumatic stress disorder and health-related quality of life among a sample of treatment- and pension-seeking deployed Canadian forces peacekeeping veterans. Canadian Journal of Psychiatry.

[b40] Roberts NP, Kitchiner NJ, Kenardy J, Bisson J (2009). Multiple session early psychological interventions for the prevention of post-traumatic stress disorder. Cochrane Database of Systematic Reviews.

[b41] Sadler AG, Booth BM, Nielson D, Doebbeling BN (2000). Health-related consequences of physical and sexual violence: women in the military. Obstetrics & Gynecology.

[b42] Schnurr PP, Hayes AF, Lunney CA, McFall M, Uddo M (2006). Longitudinal analysis of the relationship between symptoms and quality of life in veterans treated for posttraumatic stress disorder. Journal of Consulting and Clinical Psychology.

[b43] Sheehan DV, Lecrubier Y, Sheehan KH, Amorim P, Janavs J, Weiller E, Hergueta T, Baker R, Dunbar GC (1998). The Mini-International Neuropsychiatric Interview (M.I.N.I.): the development and validation of a structured diagnostic psychiatric interview for DSM-IV and ICD-10. The Journal of Clinical Psychiatry.

[b44] Stoll C, Kapfhammer HP, Rothenhäusler HB, Haller M, Briegel J, Schmidt M, Krauseneck T, Durst K, Schelling G (1999). Sensitivity and specificity of a screening test to document traumatic experiences and to diagnose post-traumatic stress disorder in ARDS patients after intensive care treatment. Intensive Care Medicine.

[b45] Try E, Morken T, Hunskår S (2008). A personal crisis support team at an accident and emergency department. Tidsskrift for Den Norske Legeforening.

[b46] Wang CH, Tsay SL, Bond AE (2005). Post-traumatic stress disorder, depression, anxiety and quality of life in patients with traffic-related injuries. Journal of Advanced Nursing.

[b47] Ware JE, Sherbourne CD (1992). The MOS 36-item Short-Form Health Survey (SF-36): I. Conceptual framework and item selection. Medical Care.

[b48] Ware JE, Kosinski M, Gandek B (2002). SF-36 Health Survey: Manual and Interpretation Guide.

[b49] Willitts M, Benzeval M, Stansfeld S (2004). Partnership history and mental health over time. Journal of Epidemiology and Community Health.

[b50] Wyrwich KW, Bullinger M, Aaronson N, Hays RD, Patrick DL, Symonds T, The Clinical Significance Consensus Meeting Group (2005). Estimating clinically significant differences in quality of life outcomes. Quality of Life Research.

[b51] Zakariassen E, Burman RA, Hunskaar S (2010). The epidemiology of medical emergency contacts outside hospitals in Norway – a prospective population based study. Scandinavian Journal of Trauma, Resuscitation and Emergency Medicine.

